# DSCAM is differentially patterned along the optic axon pathway in the developing *Xenopus* visual system and guides axon termination at the target

**DOI:** 10.1186/s13064-022-00161-9

**Published:** 2022-04-15

**Authors:** Rommel Andrew Santos, Rodrigo Del Rio, Alexander Delfin Alvarez, Gabriela Romero, Brandon Zarate Vo, Susana Cohen-Cory

**Affiliations:** Department of Neurobiology and Behavior, University of California Irvine, 2205 McGaugh Hall, Irvine, CA 92697-4550 USA

**Keywords:** DSCAM, In vivo imaging, Retina, Optic nerve, Retinal ganglion cell, Axon targeting, Optic tectum, *Xenopus laevis*

## Abstract

**Background:**

The *Xenopus* retinotectal circuit is organized topographically, where the dorsal–ventral axis of the retina maps respectively on to the ventral-dorsal axis of the tectum; axons from the nasal-temporal axis of the retina project respectively to the caudal-rostral axis of the tectum. Studies throughout the last two decades have shown that mechanisms involving molecular recognition of proper termination domains are at work guiding topographic organization. Such studies have shown that graded distribution of molecular cues is important for topographic mapping. However, the complement of molecular cues organizing topography along the developing optic nerve, and as retinal axons cross the chiasm and navigate towards and innervate their target in the tectum, remains unknown. Down syndrome cell adhesion molecule (DSCAM) has been characterized as a key molecule in axon guidance, making it a strong candidate involved in the topographic organization of retinal fibers along the optic path and at their target.

**Methods:**

Using a combination of whole-brain clearing and immunohistochemistry staining techniques we characterized DSCAM expression and the projection of ventral and dorsal retinal fibers starting from the eye, following to the optic nerve and chiasm, and into the terminal target in the optic tectum in *Xenopus laevis* tadpoles. We then assessed the effects of DSCAM on the establishment of retinotopic maps through spatially and temporally targeted DSCAM knockdown on retinal ganglion cells (RGCs) with axons innervating the optic tectum.

**Results:**

Highest expression of DSCAM was localized to the ventral posterior region of the optic nerve and chiasm; this expression pattern coincides with ventral fibers derived from ventral RGCs. Targeted downregulation of DSCAM expression on ventral RGCs affected the segregation of medial axon fibers from their dorsal counterparts within the tectal neuropil, indicating that DSCAM plays a role in retinotopic organization.

**Conclusion:**

These findings together with previous studies demonstrating cell-autonomous roles for DSCAM during the development of pre- and postsynaptic arbors in the *Xenopus* retinotectal circuit indicates that DSCAM exerts multiple roles in coordinating axon targeting and structural connectivity in the developing vertebrate visual system.

## Background

During embryonic eye development, connections from the retina to the brain are carefully arranged in a preserved spatial manner that creates a topographic map of the visual world. In the amphibian visual system, retinal ganglion cell (RGC) axons project to the tectum in a manner that mirrors the relative positioning of RGCs across the retina – effectively constructing a point-to-point representation of visual space in the brain [1–3]. The formation of precise topographic maps requires active molecular cues guiding specific axon targeting and establishing selective synaptic connections. For example, in the developing embryonic *Xenopus* visual system, dorsal retinal axons expressing high levels of Ephrin-B ligands specifically target ventral tectal regions with high EphB receptor expression via an attractive guidance mechanism [4]. Such studies demonstrate that molecular recognition of proper termination domains, often organized in matching gradient distribution, are important for topographically organizing neuronal circuits during development. Likewise, in mouse models, topographic mapping of retinal axons along the anterior–posterior axis of the superior colliculus (equivalent to the tectum in lower vertebrates) relies heavily on repulsive-mediated signaling between EphA receptors and their Ephrin-A ligands [5–7]. Disrupting the signaling gradient either by knocking out the receptor or the ligand affects topographic ordering, but not entirely [5–7]. Disruption of ephrin signaling, only to a certain extent, shifts axonal fibers posteriorly and others anteriorly [8]. Furthermore, prior to reaching the tectum, retinal axon fibers are already topographically sorted along the optic nerve where graded ephrin signaling has not been reported [9–13]. These findings suggest that ephrin signaling does not exclusively shape topography and that additional key molecules are involved.

Down Syndrome Cell Adhesion Molecule (DSCAM) has been implicated in multiple aspects of neural circuit development, modulating dendrite and axon growth in both the vertebrate and invertebrate nervous systems [14]. A specific role for DSCAM in axon growth, fasciculation and guidance is supported by a number of studies [15–19], but whether the molecule is involved in the topographic organization of retinal fibers had yet to be investigated. Multiple studies have confirmed that DSCAM is expressed by RGCs and in retinal projections along the developing mouse optic nerve [15, 20–22]. Erskine and colleagues showed that DSCAM knock out disrupted the timing at which mouse retinal axons arrived at the thalamus, suggesting that DSCAM acts as a permissive signal and mediates growth-promoting interactions that help facilitate retinal axon growth towards their target [15]. DSCAM was also shown to be involved in segregating contralateral retinal projections from ipsilateral fibers in the dLGN [20]. While these studies did not directly test DSCAM’s involvement in organizing retinal topography, they indicate that DSCAM may contribute to the specificity of axonal wiring within the target. Previous work from our laboratory showed that in *Xenopus*, DSCAM acts as a permissive signal that facilitates axon arbor growth once RGC axons reach their target in the optic tectum [23]. Here, we used the *Xenopus* tadpole visual system to further examine potential roles for DSCAM in establishing retinotopic order as axons travel towards and establish synaptic connections at their target. We observed differential DSCAM expression along the ventral and posterior regions of the optic nerve and chiasma, indicating that subpopulations of retinal fibers differentially express DSCAM as they navigate the optic path. By tracing the projection of ventral and dorsal retinal fibers as they exit the eye into the optic nerve and chiasm in fixed tadpoles, and imaging in vivo retinal axon arbors in the *Xenopus* optic tectum with altered DSCAM expression, we provide evidence that DSCAM is expressed in a topographic manner along the optic nerve and chiasm and affects the segregation of axon fibers derived from ventral RGCs from neighboring axon fibers from dorsal RGCs within the tectal neuropil.

## Methods

### Animals

*Xenopus* *laevis* embryos were obtained via natural mating between adult male and female frogs. Adult frogs of both sexes were primed with human chorionic gonadotropin (10,000 units; Millipore Sigma) before natural mating. Embryos and tadpoles were raised in rearing solution (60 mM NaCl, 0.67 mM KCl, 0.34 mM Ca(NO_3_)_2_, 0.83 mM MgSO_4_, 10 mM HEPES, pH 7.4, and 40 mg/L gentamycin) supplemented with 0.001% phenylthiocarbamide (PTU) to prevent melanocyte pigmentation. All embryos were anesthetized during experimental manipulations with 0.05% tricane methanesulfonate (Finquel; Argent Laboratories, Redmond, WA). Staging of embryos was performed according to Nieuwkoop and Faber [24]. Animal procedures were approved by the Institutional Animal Care and Use Committee of the University of California, Irvine (Animal Welfare Assurance Number A341601).

### Immunohistochemistry

Stage 45–46 *Xenopus laevis* tadpoles were euthanized with tricaine methanesulfonate (Finquel MS-222) and fixed in 4% paraformaldehyde in 1 × PBS, pH 7.5, for 4 h. Tadpoles were cryoprotected in 30% sucrose for 1 h at room temperature, and embedded in OCT compound (Sakura Finetek, Torrance, CA, USA). 40-μm coronal and horizontal cryostat sections were obtained and washed with phosphate buffered saline + 0.01% Tween-20 (1 × PBST) 3 times, 5 min each. Sections were then blocked, for 1 h using 10% normal goat serum (Antibodies Incorporated) in 1 × PBST. Blocking solution was removed and sections were incubated overnight with an antibody against the middle region of human DSCAM (rabbit polyclonal, 1:1000 dilution; Aviva System, San Diego, CA, USA) and 3A10 mouse anti-neurofilament-associated protein antibody (1:2000; Developmental Studies Hybridoma Bank) in blocking solution (2% normal goat serum in PBST). Brain tissues were washed then incubated in goat anti-rabbit Alexa 568 and goat anti-mouse Alexa 488 secondary antibodies (1:500 dilution; Invitrogen, Eugene, OR, USA). Sections were washed prior to being stained with DAPI. Tissue samples were imaged using a LSM780 confocal microscope (Zeiss) or with a Nikon E800 epifluorescence microscope equipped with a Zyla sCMOS camera (Andor). The mean fluorescence intensity of DSCAM and 3A10 antibody staining was measured using a circular region tool on the MetaMorph imaging software (Molecular Devices, San Jose, CA). Repeated measurements were made using a defined circular region of interest (ROI; 3.5 µm diameter) tool at various anatomical locations of the sample being analyzed. Data for each ROI was normalized to the mean fluorescence intensity of each fluorescence channel (Alexa 568 and Alexa 488) for the sample being analyzed (optic nerve, chiasm, neuropil, etc.) in each tadpole sample and is expressed as percent of the mean. Statistical analysis was performed with at least two independent cryostat sections from each tadpole with four tadpoles used per parameter analyzed.

### Whole brain clearing

A *Xenopus*-Fast Clearing Technique (X-FaCT) was performed as described in the protocol by Affaticati and colleagues [25] to reduce light scattering throughout the brain tissue. In brief, stage 45 to 46 tadpoles were euthanized with Finquel and fixed in 4% paraformaldehyde in 1 × PBST overnight. Tadpoles were washed in 1 × PBST and whole heads were dissected. Tissues were first placed in pre-incubation solution 0.5 × SSC (150 mM NaCl, 15 mM sodium citrate, pH 7.2), 0.1% Tween 20, and were then incubated in depigmentation solution (5% formamide, 0.5 × SSC, 3% H_2_O_2_) to remove melanocyte pigmentation. Samples were transferred into a 2 mL glass vial and were blocked for 4 h at room temp. To visualize both DSCAM immunoreactivity and axon bundles in the tadpole’s head, tissues were incubated in DSCAM rabbit polyclonal (1:500; Aviva System) and 3A10 mouse anti-neurofilament-associated protein antibody (1:500; Developmental Studies Hybridoma Bank) in 10% DMSO, 1% Triton X-100 in 1 × PBST. Goat anti-rabbit Alexa 568 and goat anti-mouse Alexa 488 antibodies (both at 1:500; Invitrogen) were used as secondary antibodies, respectively. To reduce light scattering throughout brain and head tissues, samples were submerged in a fructose–based high–refractive index solution (1.45) at room temp overnight. Cleared samples were imaged using a LSM780 confocal microscope (Zeiss). Fluorescence intensity for DSCAM and 3A10 immunostaining was measured in individual confocal planes using MetaMorph’s circular region tool. Repeated measurements were made using a defined circular region of interest (ROI; 10 µm diameter) tool at separate locations within the midbrain neuropil and optic tract of the sample being analyzed. Data for each ROI was normalized to the mean fluorescence intensity per channel (Alexa 568 and Alexa 488) and expressed as percent of the mean. Sample size was at least three independent confocal z-sections from each tadpole with four tadpoles used for the statistical analysis. Statistical analysis was by two-way ANOVA.

### Labeling retinal ganglion cell axons

To visualize retinotopic organization, ventral and dorsal RGCs axons were labeled by direct retinal electroporation following a similar protocol developed by Haas and colleagues [26]. Tadpoles at stage 45 were anesthetized in diluted tricaine methanesulfonate. A custom-made trench, to hold the head of a stage 45 tadpole, was carved out in sylgard (Silicone Elastomer Kit). In the trench, a single embryo was placed laterally on their side and a standard size harp slice grid (ALA Scientific Instruments) was used to hold the embryo in place. The tadpole’s right eye was positioned and made available for electroporation. Lissamine-tagged standard control morpholino oligonucleotides (Gene Tools) were electroporated into the ventral quadrant of the retina to label ventral retinal axons, followed by electroporation of fluorescein-tagged control morpholinos or Alexa Fluor 488 fixable dextran (10,000 MW, Invitrogen) into the dorsal quadrant of the retina to label dorsal axon fibers. Fluorescein-tagged control morpholinos were used for histology because they labeled axon fibers better; Alexa 488 fixable dextran labeled axon arbors better for in vivo imaging.

To alter DSCAM levels in ventral RGCs, a morpholino antisense oligonucleotide (MO) targeting *Xenopus* *laevis* *Dscam* mRNA was designed with the sequence 5′-ACATATAAGACTTCGACAGAGACGT-3′. The specificity of the MO to downregulate DSCAM was demonstrated in our previous studies by injecting the *Xenopus*-specific DSCAM MO into a single blastomere of 2-cell or 4-cell stage embryos and confirming changes in expression by western blot as well as by immunostaining tadpoles with antibodies to DSCAM at different developmental stages [23]. Individual reagents were loaded into an aluminosilicate glass electrode (with filament; AF100–64–10, 1.00 mm, 0.64 mm, 10 cm) equipped with a silver wire connected to a Grass SD9 electrical stimulator. An external ground wire, connected to the stimulator, was placed in the sylgard trench dish holding the anesthetized tadpole. For lissamine-tagged MO electroporation, repeated currents were delivered at 200 Hz, 2 ms delay, 2 ms duration, 20 V until ventral RGCs were labeled. Fluorescein-tagged control MOs or Alexa 488 fixable dextran was delivered at 200 Hz, 4 ms delay, 4 ms duration, 40 V until dorsal RGCs were stained. Axon arbors in anesthetized tadpoles were imaged in vivo with a Nikon PCM2000 laser-scanning confocal microscope equipped with Argon and HeNe lasers. We quantified, blind to treatment, the territory occupied by laterally-projecting arbors (derived from dorsal RGCs) and the medially-projecting arbors (derived from ventral RGCs) using MetaMorph. The area occupied by the ventral or dorsal RGC axon terminals was measured, in pixels, by creating a ROI (polygon) surrounding the fluorescent axon arbors from the first branch point to the terminal tips of the arbors in the unmerged red or green images. The area of overlap between the two polygon areas was then measured after merging the images corresponding to the two channels. Data was normalized across tadpole samples by calculating, in percent, the area of overlap with respect to the medial (red fluorescence), lateral (green fluorescence) and medial + lateral (red and green fluorescence) axons. Unpaired t-tests were used for statistical analysis as described previously [27]. Data results were considered significant as follows: *p ≤ 0.05, ***p ≤ 0.001, ****p ≤ 0.0001.

## Results

### Specific expression of DSCAM in the developing *Xenopus* optic nerve, chiasm, and tectum

**Fig. 1:**
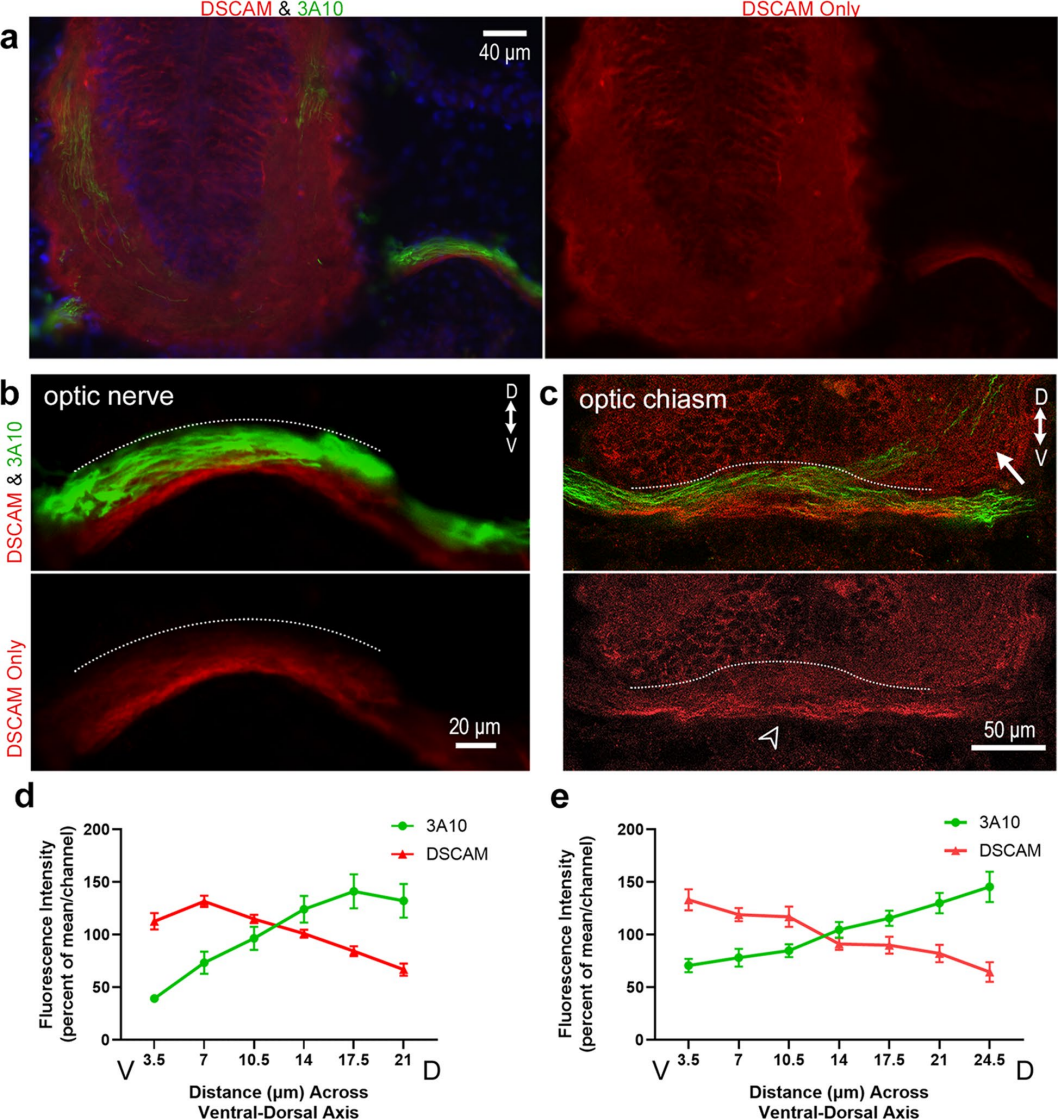
Distribution of DSCAM in a ventral-to-dorsal gradient along the optic nerve and chiasm of stage 45/46 *Xenopus* tadpoles. **a** Coronal section of the *Xenopus* tectum and optic nerve immunostained with DSCAM (red; right and left panels) and 3A10 anti-neurofilament (green; left panel overlay) antibodies. **b** Higher magnification image of the optic nerve shows high DSCAM immunoreactivity ventrally along the optic nerve bundle, while 3A10 antibody preferentially stains fibers along the dorsal region of the optic nerve (overlay; *white dotted line*). **c** Coronal section at the level of the optic chiasm (*white arrowhead*), shows DSCAM immunoreactivity localized to fibers at the ventral base of the chiasm (*white arrowhead*), while 3A10 immunopositive retinal fibers cross the optic chiasm more dorsally (*white dotted line*). Note the more diffused, punctate DSCAM immunoreactivity in the brain at the level of the optic tract (*arrow*) where RGC axons begin to defasciculate and project contralaterally past the chiasm. **d**, **e** The average fluorescence intensity of DSCAM and 3A10 immunoreactivity was measured along the ventrodorsal axis of the optic nerve and optic chiasm using a 3.5 μm diameter ROI using MetaMorph. Six or seven measurements using the circular region tool were obtained along the ventrodorsal axis, with five sets of measurements obtained at distinct locations along the optic nerve or chiasm (*n* = 4 tadpoles, with at least two tissue sections per tadpole). **d** Plotting mean fluorescence intensity (normalized per channel and per sample) along the optic nerve revealed a high-ventral to low-dorsal distribution of DSCAM immunoreactivity while 3A10 immunoreactivity was higher dorsally (difference for *Distance p* = 0.011; *Distance x Fluorescent Channel p* ≤ 0.001; two-way ANOVA). **e** Quantification of the mean fluorescence intensity shows a high-ventral to low-dorsal distribution of DSCAM immunoreactivity at the optic chiasm (difference for *Distance p* = 0.003; *Distance x Fluorescent Channel p* ≤ 0.001; two-way ANOVA). Scale bars: 40 μm for a; 20 μm for b, 50 μm c

Previous work from our laboratory showed that DSCAM immunoreactivity localizes to the membrane of neurons in the RGC layer within the retina and neurons in the optic tectum in *Xenopus* tadpoles at stage 45 [23]. Without permeabilization, punctate DSCAM immunoreactivity was localized to the tectal neuropil where retinal axons and tectal neuron dendrites establish functional synaptic connections at this stage. These observations led us to further characterize DSCAM expression patterns across the *Xenopus* visual system as a means to inform us about its roles in the structural development of retinotectal circuits. For these experiments, we permeabilized tissues and co-immunostained sections of stage 45 to 46 tadpoles with antibodies to DSCAM (as in our previous study [23]) together with the anti-neurofilament protein antibody (Mab 3A10) that has been shown to label a subset of retinal axons [28]. As observed on coronal tissue sections, DSCAM immunoreactivity was highly localized to the ventral region of the optic nerve (Fig. 1a, b). Fluorescence intensity for both DSCAM and 3A10 immunoreactivity was measured across the ventral-dorsal axis of the optic nerve bundle (measurements started at the ventral side of the optic nerve then continued to the dorsal side as marked by the white dotted line on Fig. 1b). DSCAM immunofluorescent signal progressively decreased as measurements were obtained along the dorsal regions of the optic nerve. We normalized and plotted fluorescence intensity on a graph with the x-axis representing regions along the ventral to dorsal portions of the optic nerve (Fig. 1d; *n* = 4 tadpoles). This analysis quantitatively confirmed a high-ventral to low-dorsal graded pattern of DSCAM expression. In contrast, 3A10 immunoreactivity strongly localized to the dorsal region of the optic nerve (border noted by white dotted line on Fig. 1b) with a lower intensity signal observed along the ventral side. Quantitatively, 3A10 immunofluorescence was lower ventral and higher dorsal (Fig. 1d), a distribution that appeared to be an inverse of the high-ventral to low-dorsal DSCAM immunoreactivity pattern. Similarly, analysis of retinal axons as they crossed the midline at the optic chiasm (white arrowhead, Fig. 1c) showed that DSCAM immunoreactivity strongly localized at the ventral base of the chiasm. Here again, the intensity of DSCAM immunostaining gradually decreased towards the more dorsal areas of the optic chiasm (white dotted line, Fig. 1c, e), while 3A10 immunoreactivity was higher dorsally along the optic chiasm (Fig. 1c, e). DSCAM immunoreactivity became less distinguishable in axon fibers projecting contralaterally along the optic tract past the chiasm (arrow, Fig. 1c).

**Fig. 2:**
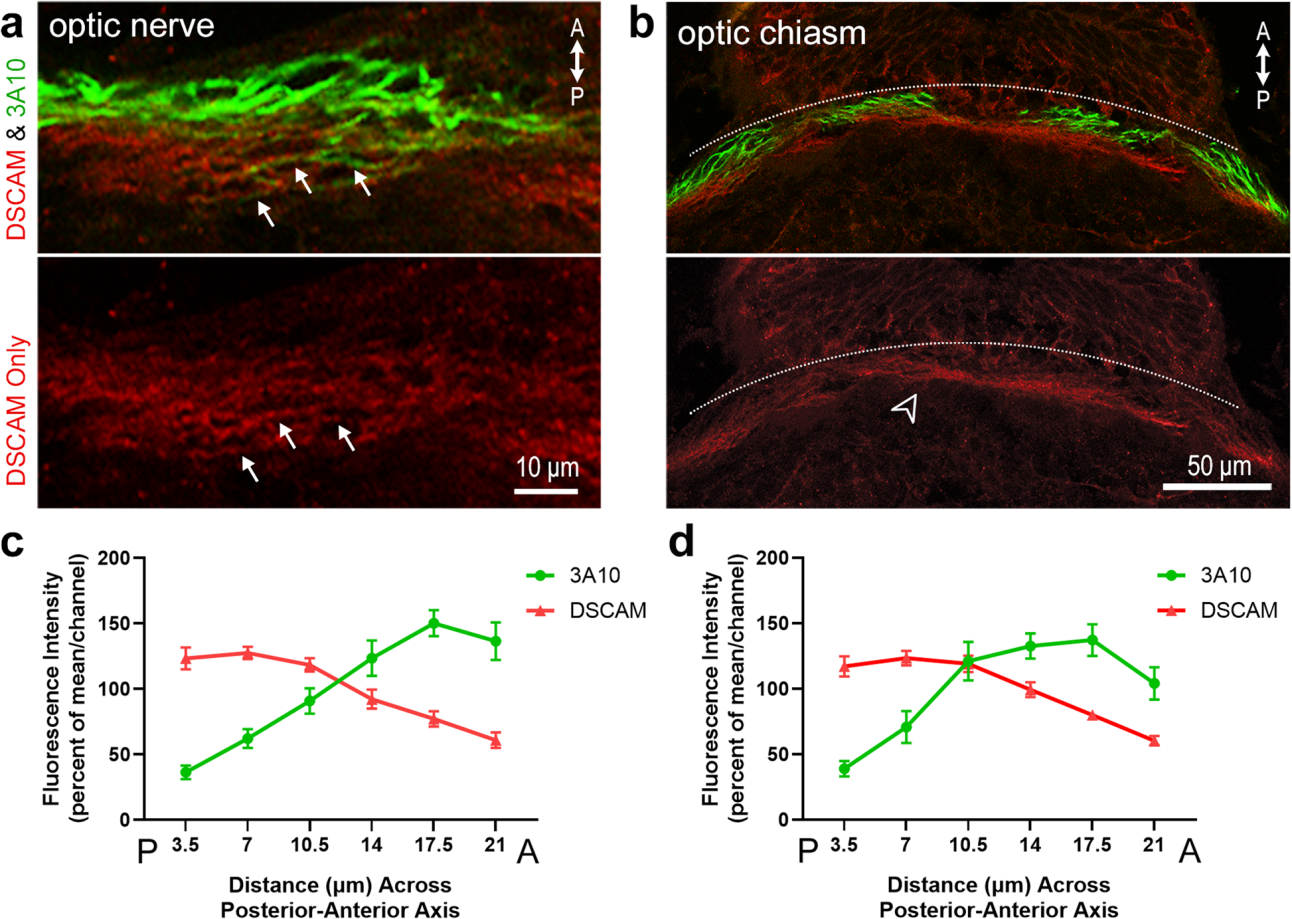
Distribution of DSCAM in the optic nerve and chiasm along the posterior-to-anterior axis of stage 45/46 *Xenopus* tadpoles. **a** High magnification horizontal section at the level of the optic nerve shows differential distribution of DSCAM immunoreactivity (red) along retinal axon fibers. A gradual distribution of DSCAM immunoreactive fibers is observed from posterior to anterior while most 3A10 immunopositive fibers localize more anteriorly. White arrows identify a subset of axon fibers within the optic nerve that are immunopositive for both DSCAM and 3A10. **b** Horizontal section at the level of the optic chiasm imaged by confocal microscopy (stitched tiled scan) shows DSCAM immunoreactivity in fibers that organize caudally (posterior; empty white arrowhead) to most 3A10 immunopositive fibers. **c, d** Fluorescence intensity of DSCAM (red) and 3A10 (green) immunoreactivity was measured using a 3.5 μm ROI at six positions along the posterior to anterior axis tool as for Fig. 1, with five sets of measurements made at distinct locations along the optic nerve or optic chiasm (*n* = 4 tadpoles, with at least two tissue sections per tadpole). For each tissue sample, fluorescence intensity was normalized to the mean fluorescence intensity for each channel and is shown as percent of the mean with the x-axis designating the optic nerve or optic chiasm from the posterior to anterior regions. **c** A high-posterior to low-anterior distribution of DSCAM fluorescent signal is found along the width of the optic nerve, while 3A10 fluorescent signal is higher in the anterior portion of the optic nerve (difference for *Distance p* = 0.008; *Distance x Fluorescent Channel p* ≤ 0.0001; two-way ANOVA). **d** DSCAM and 3A10 fluorescence showed high-posterior to low-anterior DSCAM immunoreactivity within the optic chiasm, while 3A10 immunofluorescent signal was low posterior and increased anteriorly (difference for *Distance* *p* = 0.002; *Distance x Fluorescent Channel p* ≤ 0.0001; two-way ANOVA). Scale bars: 10 μm for a; 50 μm c

Having encountered a differential distribution of DSCAM along the ventrodorsal axis of the optic nerve and chiasm, we further characterized DSCAM expression along the posterior-anterior axis. In horizontal tissue sections, we found higher DSCAM immunofluorescence intensity specifically at the posterior region of the optic nerve bundle (Fig. 2a) and optic chiasm (white arrowhead, Fig. 2b). DSCAM immunofluorescence intensity was lower at the anterior portions of the optic nerve and chiasm compared to the posterior side, indicating a high-posterior to low-anterior pattern of DSCAM expression (Fig. 2 c, d). Immunostaining with the 3A10 antibody showed an inverse pattern, where the fluorescence distribution of 3A10 staining appeared lower posterior and higher anterior at both the optic nerve and chiasm (Fig. 2 a-d). It is important to note that DSCAM and 3A10 immunoreactivity co-localized along a number of fibers both in the optic nerve and chiasm (white arrows, Fig. 2a), confirming that the DSCAM immunostaining identified RGC axon fibers. These results suggest that a subpopulation of RGC axon fibers differentially relies on DSCAM as a potential mechanism to navigate the optic nerve pathway and cross the optic chiasm.

**Fig. 3:**
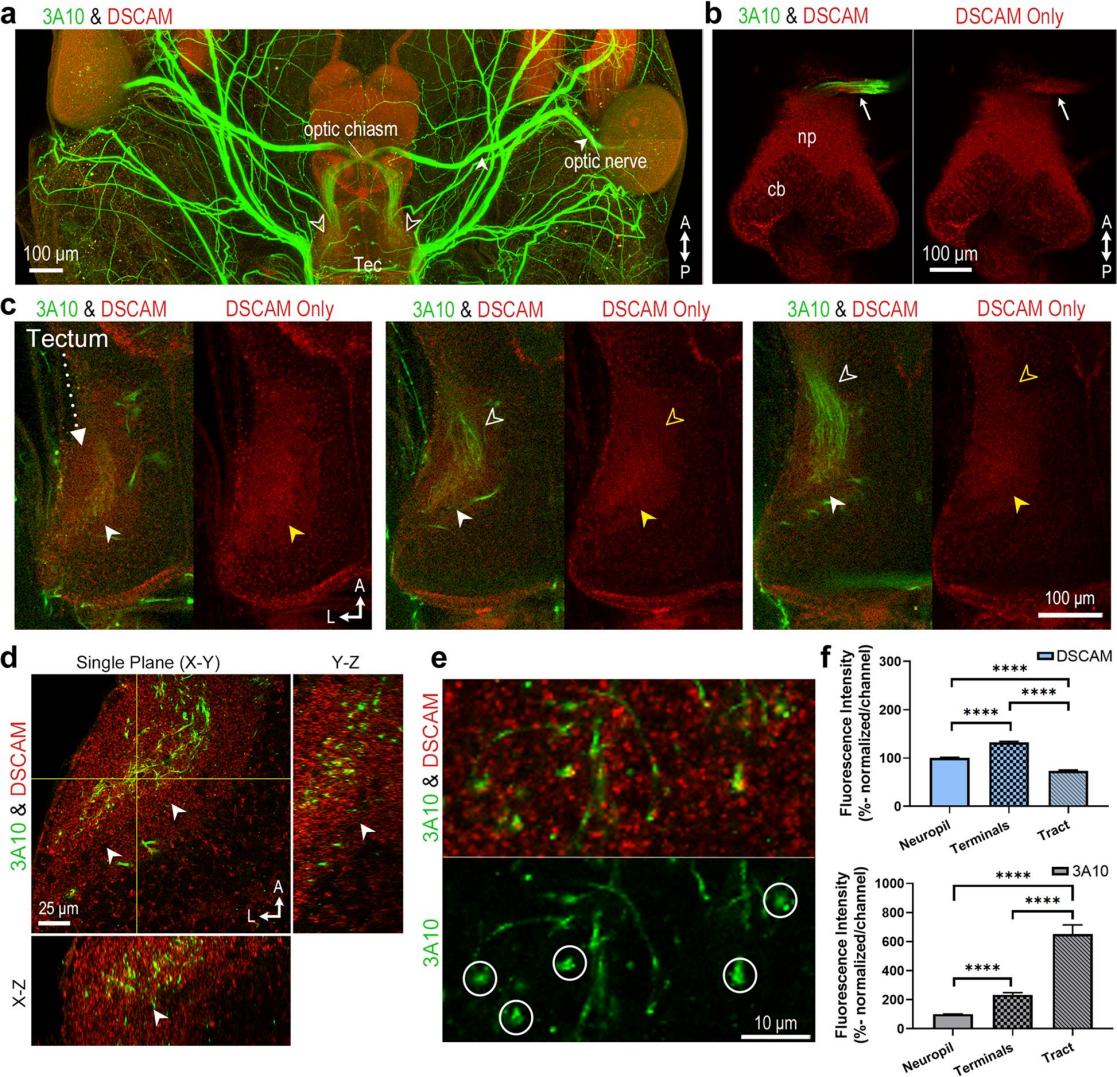
Visualizing DSCAM expression in cleared *Xenopus* brain tissues. **a**, **b** Whole tissue clearing followed by immunostaining was used to characterize DSCAM expression in intact *Xenopus laevis* tadpoles. (**a**) Low-magnification confocal imaging (tiled scan) of a tadpole head shows DSCAM immunoreactivity (red), and 3A10 antibody co-immunostaining (green). Optic nerve fibers (*solid white arrowhead*) and RGC axon terminals within the tectal neuropil (*empty white arrowheads*) are visualized by the 3A10 immunostaining. In addition to the optic nerve, the 3A10 antibody stains axonal fibers in sensory and motor cranial nerves. **b** Individual confocal plane from a z-stack of a brain (imaged ventrally) shows differential distribution of DSCAM and 3A10 immunoreactivity along the optic nerve as it enters the optic chiasm (*arrow*). DSCAM immunoreactivity also localizes to cell bodies (*cb*) and neuropil (*np*). **c** Individual confocal planes from horizontal z-stacks further illustrate co-localization of DSCAM and 3A10 immunoreactivity in the midbrain neuropil (from dorsal-left to ventral-right). Stronger DSCAM immunoreactivity is observed on the dorsal-most portion of the tectum, where axon terminals extensively branch (*solid white* and *yellow arrowheads*). The 3A10 antibody staining also reveals RGC axon fibers as they enter the midbrain (*empty arrowheads*). **d** Higher magnification confocal images of dissected brains illustrate strong punctate DSCAM immunoreactivity in the area of the neuropil where axon terminals localize, as identified by the 3A10 immunostaining (*arrowheads*; yellow lines indicate location of x–z and y–z orthogonal planes, thickness of sample imaged was 85 µm). **e** Magnified, one-micron z-section of the cleared brain in ***d*** reveals coincident punctate DSCAM immunostaining (red) in 3A10-immunolabeled growth cones (green). **f** The fluorescence intensity of DSCAM (*top graph*) and 3A10 (*bottom graph*) immunostaining was measured in whole brain tissues using a 10 μm circular ROI tool and analyzed using MetaMorph, with ten measurements obtained across ten regions each at the level of the lateral ***neuropil***, axon ***terminals***, and axon ***tract***. Measurements were taken from both brain hemispheres equally (*n* = 4 tadpoles). Data is normalized to the mean fluorescence intensity per channel in the lateral neuropil. Error bars indicate S.E.M. **** *p* ≤ 0.0001. Scale bars: 100 μm for **a**, **b** and **c**; 25 μm for **d**; 10 μm for **e**

To further determine if axonal arbors terminating and branching in the optic tectum express DSCAM in a pattern similar to the high-ventral to low-dorsal pattern found along the optic nerve and chiasm, we performed immunostaining of whole brain cleared tissues to preserve the structural layout of axonal tracts and arbors innervating the tectum. Compared to brain sectioning, brain clearing is a powerful technique that permits obtaining a novel three-dimensional perspective of any potential gradient pattern of DSCAM expression within the intact tectum [25]. Cleared tissue samples of stage 45 to 46 tadpoles were immunostained with DSCAM and 3A10 antibodies (Fig. 3). This technique revealed that the optic nerve (solid white arrowhead, Fig. 3a), as well as sensory and motor cranial nerves throughout the tadpole head were immunostained by the 3A10 antibody (Fig. 3a). Individual confocal planes of cleared brain tissues imaged ventrally showed that DSCAM immunoreactivity was higher posteriorly along the optic nerve as it enters the optic chiasm (arrow, Fig. 3b). At the optic tectal neuropil, strongest DSCAM immunoreactivity coincided with axon terminals labeled with the 3A10 antibody (empty white arrowheads at the tectum, Fig. 3a). When we examined individual horizontal z-stacks, we observed that within the neuropil, DSCAM immunoreactivity was higher at the level where RGC axons terminate as revealed by the 3A10 co-immunostaining (solid white and yellow arrowheads; Fig. 3c, left and middle panels), and lower at fasciculated axon bundle tracts as they enter the neuropil (empty white and yellow arrowheads, Fig. 3c, middle and right panels). Viewing single confocal orthogonal planes of cleared, dissected brain tissues confirmed that strong DSCAM immunoreactivity localized to the area of the neuropil with the 3A10 retinal axon marker (solid white and yellow arrows, Fig. 3d). Single plane analysis of high magnification confocal z-stacks also revealed DSCAM punctate staining on 3A10-stained growth cones in axons terminals (Fig. 3e). When compared to the area devoid of retinal fibers, quantitatively DSCAM fluorescence intensity was significantly higher within the area of the tectal neuropil where 3A10-immunolabeled RGC axon terminals localize (Fig. 3f). DSCAM immunoreactivity was also higher within the area where RGC axons terminate when compared to the more anterior optic tract where the 3A10-immunostained axon fibers path-find (Fig. 3f).

**Fig. 4:**
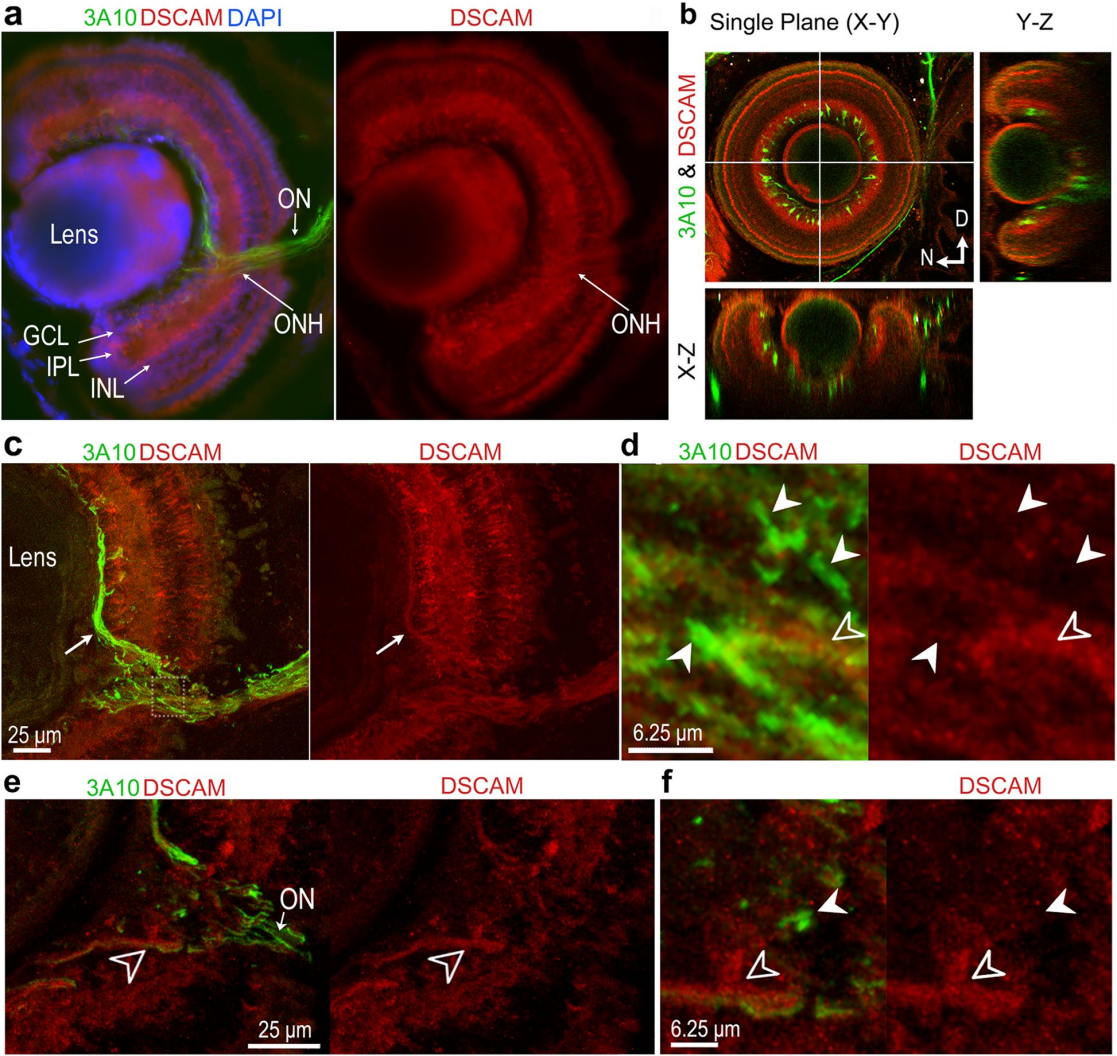
Differential expression of DSCAM by RGC axon fibers in retina. **a** Epifluorescent image of a coronal section of a stage 45 tadpole eye immunostained with DSCAM (red; right panel DSCAM only) and 3A10 antibodies (green) shows strong DSCAM immunoreactivity in the in the ganglion cell layer (GCL), inner plexiform layer (IPL) and Inner nuclear layer (INL). 3A10 immunopositive cell bodies are confined to the GCL, adjacent to the IPL. **b** Frontal and orthogonal confocal views of an eye in a cleared *Xenopus* tadpole head immunostained with DSCAM (red) and 3A10 anti-neurofilament (green) antibodies shows strong DSCAM immunoreactivity in the GCL and IPL and differential distribution of 3A10 immunoreactivity in a subset of cells in the GCL (lines indicate location of x–z and y–z orthogonal planes, thickness of sample imaged was 296 µm). **c** Confocal images of a tadpole eye illustrate DSCAM and 3A10 immunoreactivity at the level of the optic fiber layer (*arrow*) within the retina and in the optic nerve head (*box*). **d** The magnified images (box in ***c***) show a subset of 3A10-immunopositive axon fibers (*white arrowheads*) that do not express DSCAM (left panel, 3A10 and DSCAM overlap; right panel, DSCAM only). **e**, **f** A single confocal plane shows coincident immunoreactivity for DSCAM and 3A10 (*empty arrowheads*) in ventral fibers along the optic nerve head, as also illustrated in ***d*** (*empty arrowhead*). Scale bars are as shown for each image

To determine whether the patterns of DSCAM expression in the optic nerve, chiasma and in the optic tectum correspond with differential DSCAM expression within the retina, we analyzed retinas of stage 45 tadpoles in cleared intact tissues and in cryostat sections immunostained with DSCAM and the 3A10 antibodies (Fig. 4). As previously shown [23], DSCAM immunoreactivity was observed in the ganglion cell layer (GCL), inner plexiform layer (IPL) and inner nuclear layer (INL) of the *Xenopus* retina, with punctate DSCAM expression found around cell bodies within the GCL (Fig. 4a). Because no differential expression of DSCAM could be discerned within the layers of the retina between the dorsal or ventral axis (Fig. 4a, see also [23]), it is possible that DSCAM function along optic axon nerve bundles and axon terminals is separate or independent from its function within the local retinal circuit [22, 23, 29–31]. In the retina, the majority of cell bodies immunostained with the 3A10 antibody localized to the GCL, adjacent to the IPL. However, as observed both in coronal sections and in cleared intact eyes, not all RGCs were immunopositive for 3A10 (Fig. 4a-c) indicating that subsets of RGCs differentially express the neurofilament-associated proteins recognized by the 3A10 antibody. Analysis of confocal sections of retinas co-immunostained with DSCAM and 3A10 antibodies showed that some axon fibers exiting the eye along the optic fiber layer (Fig. 4c; arrow) and the optic nerve head (Fig. 4c, box, Fig. 4d) were immunopositive for both DSCAM and 3A10 (Fig. 4d; empty arrowheads), although stronger DSCAM immunoreactivity localized to axon fibers with weaker 3A10 immunoreactivity (Fig. 4e; empty arrowheads). However, a number of 3A10 positive fibers did not stain for DSCAM (white arrowheads; Fig. 4d, f). Together, these observations show a differential pattern of expression of DSCAM by RGC axons as they exit the eye and reveal that different subsets of RGCs, including those that differentially express DSCAM and neurofilament-associated proteins recognized by the 3A10 antibody, appear to organize in distinct topographic order as they navigate along their path to their target in the optic tectum.

### Dorsoventral axon sorting in the *Xenopus* retinotectal system and DSCAM effects on topographic segregation at the optic tectum

**Fig. 5:**
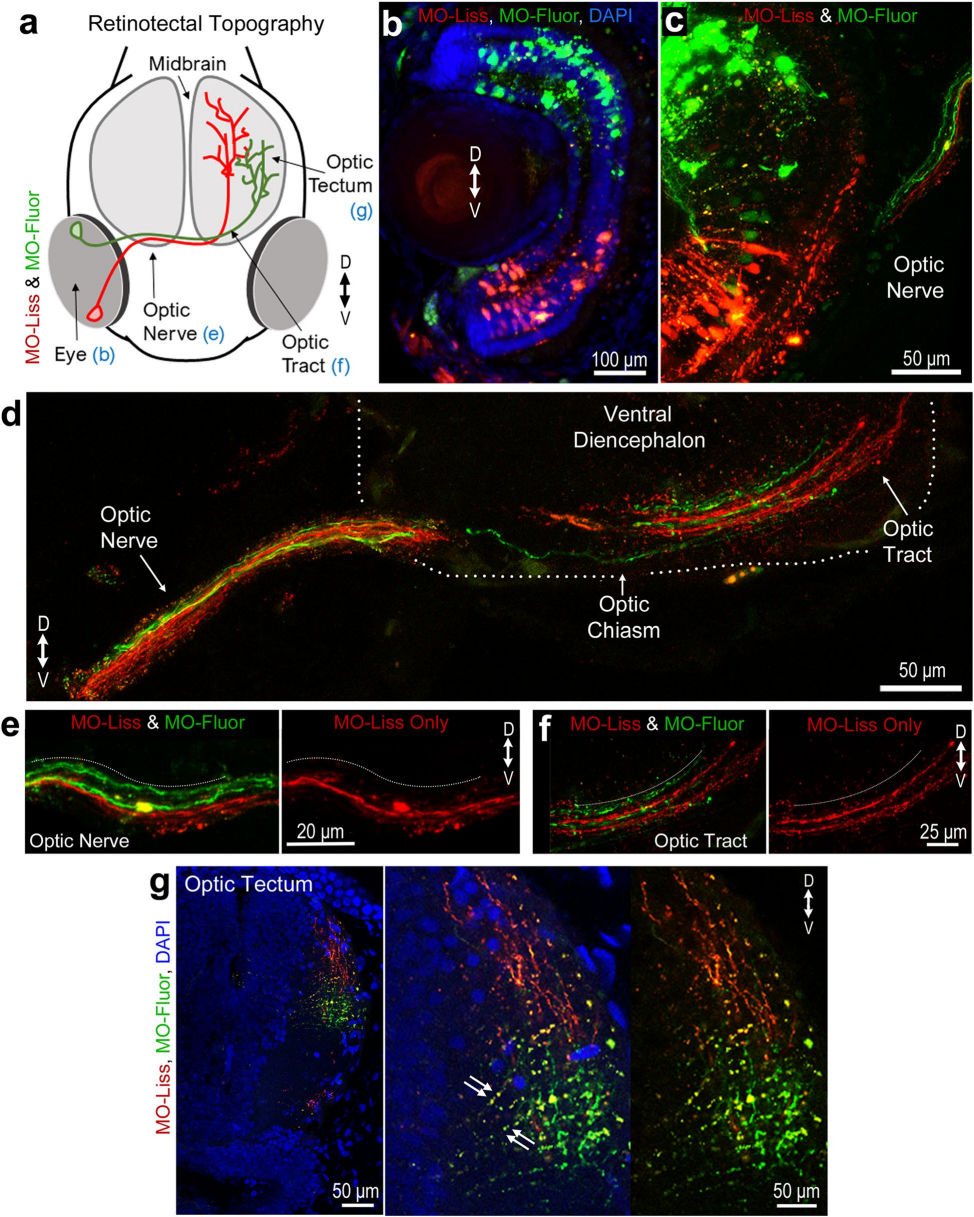
Topographic organization of retinal axon fibers along the developing *Xenopus* retinotectal path. **a** Schematic representation of the developing tadpole visual system and of experimental design. **b** Coronal section of a stage 46 tadpole eye shows localization of a lissamine-tagged control MO (red) after electroporation into the ventral half of the retina and fluorescein-tagged control MO into the dorsal half (green). **c**, **d** High magnification confocal images show the trajectories of lissamine-tagged RGCs axon fibers and fluorescein-tagged control MO labeled axon fibers as they exit the eye **c** and along the optic nerve, chiasm, and optic tract **d.** Note the topography of RGCs in the eye and their spatial arrangement along the optic nerve and chiasm, where axon fibers from ventral RGCs labeled by the lissamine tag travel along the ventral side of the optic nerve and chiasm while axons of RGCs labeled fluorescein-tagged control MO travel along the dorsal side of the optic nerve. **d**, **e**, **f** Lissamine MO-labeled axon fibers that were originally positioned on the ventral side of the optic nerve are positioned more dorsally after crossing the optic chiasm. In contrast, fluorescein MO-labeled axon fibers that originate in the dorsal portion of the retina shift more ventrally. In the stitched tiled image in *d*, the ventral tissue border of the brain is demarcated by the dashed line. **g** At the optic tectum, the lissamine-labeled RGC axon fibers localize to the dorsal branch while the fluorescein-tagged control MO-labeled axon fibers localize ventrally. Small double arrows in the magnified image point to fluorescent debris picked by glial cells. Scale bars: 100 μm for **b**; 50 μm for **c**, **d**, and **g**; 20 μm for **e**; 25 μm for **f**

A graded distribution of molecular cues has largely been implicated in topographic mapping. Based on its differential distribution, it is likely that DSCAM collaborates with other guidance and cell adhesion molecules in the topographic organization of axon retinal fibers at multiple points along their path and/or at their target [32]. Indeed, analysis of a mouse model of Down syndrome showed that DSCAM regulates eye-specific segregation of retinogeniculate projections at the target, in the dorsal lateral geniculate nucleus [20]. Thus, to explore whether DSCAM is directly involved in retinotopic organization in the *Xenopus* optic tectum, we first characterized the projection and ordering of ventral and dorsal retinal fibers as they travel from the eye through the chiasm and into the brain (as depicted schematically in Fig. 5a). A scrambled control fluorescein-tagged MO (to serve as a green fluorescent marker) and a control lissamine-tagged MO (red fluorescent marker) were electroporated separately to label dorsal and ventral RGCs, respectively (Fig. 5b, c). Although fluorescently tagged morpholinos do not stain and reveal the entire complexity of RGC axon terminal arbors, their transport along the axons to the tip of the terminals served as a reliable marker to topographically target and label RGCs and their axons. Our results show that ventral RGCs project axon fibers that are positioned along the ventral portion of the optic nerve, while dorsal RGCs send axon fibers along the dorsal region of the optic nerve (Fig. 5c, d, e). As axons of both ventral and dorsal RGCs enter and cross the chiasm and turn contralaterally into the tectum, we observed a shifting of fiber arrangement, with lissamine MO-labeled axon fibers that were originally positioned on the ventral side of the optic nerve intermixing and positioning more dorsally after crossing the chiasm (Fig. 5d, f). This inverted projection was also observed for the fluorescein MO-labeled axon fibers that originate in the dorsal portion of the retina, shifting more ventrally (Fig. 5f). A complete inverted arrangement was observed for axons as they innervate the tectum, with ventral RGC axons entering the tectum through the dorsal branch and dorsal RGC axons projecting ventrally within the tectum (Fig. 5g) in agreement with previous studies [33, 34]. Thus, our analysis of the topographic organization of dorsal and ventral RGC axons as they travel from the optic nerve to their target complements our immunohistochemical data and indicates that specific DSCAM expression along the ventral portion of the optic nerve would coincide with axon fibers traveling on the ventral side of the optic nerve pathway prior to crossing towards the tectum (Figs. 1b, c and 3b).

**Fig. 6:**
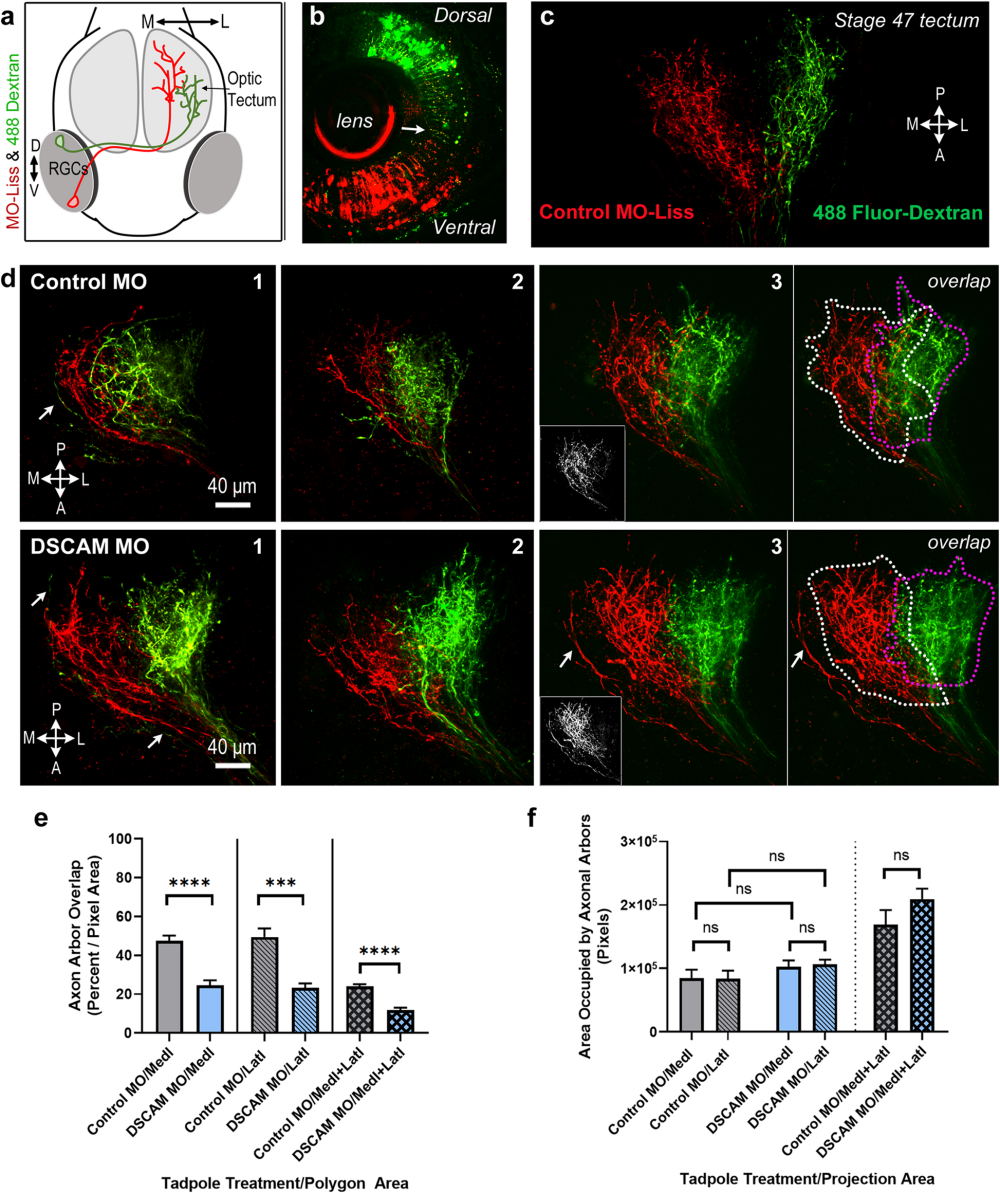
DSCAM impacts the topographic organization of ventral RGC axon fibers branching at the target. **a** Schematic illustrates the sequential electroporation of ventral RGCs with lissamine-tagged control or DSCAM MO, and dorsal RGCs with Alexa Fluor 488-dextran. **b** Coronal section of a stage 46 tadpole eye shows the distribution of the lissamine-tagged MO and Alexa Fluor 488-dextran after electroporation. Arrow points to fluorescent debris picked by glial cells. **c** Confocal projection of axon terminals of a tadpole transfected at stage 47 and imaged in vivo 48 h after transfection illustrates the organization of retinal fibers along the medial to lateral axis. Control MO lissamine-tagged axons (red) from ventral RGC terminate medially (M) within the tectal neuropil while Alexa Fluor 488 dextran-labeled axons (green) from dorsal RGCs terminate more laterally (L) with little to no overlap. **d** Confocal projections of axon terminals from three sample tadpoles with ventral RGCs transfected with lissamine-tagged Control MO or DSCAM MO (red) at stage 46 and imaged 48 h after transfection show some degree of overlap between RGC axons labeled with Alexa Fluor 488 dextran (green) and MO-transfected RGC axons (red). Single channel fluorescent signals for arbors of ventral RGC transfected with Control MO and DSCAM MO are shown in the insets. Transfection of DSCAM MO in ventral RGCs resulted in their axons projecting medially (red) but with less overlap than those in Control MO transfected tadpoles. **e**, **f** Quantitative analysis of the territory occupied by dorsal and ventral RGCs axons within the tectal neuropil in tadpoles transfected with either DSCAM MO or Control MO and imaged 48 h later. The area occupied by the ventral or dorsal RGC axon terminals was measured by separately creating a polygon surrounding the first branch point and the terminal tips of the lissamine-labeled arbors (red only; see white dashed line in ***d***) or the Alexa 488-labeled arbors (green only; see magenta dashed lines in ***d***). Isolated, unbranched axons that project medially (arrows in ***d***) were not included in the analysis. **e** A significant difference in the area of arbor overlap is observed when comparing axon arbors from ventral RGCs that project medially in DSCAM MO vs Control MO treated tadpoles. Data was normalized across tadpole samples by calculating, in percent, the area of overlap with respect to the region occupied by the medial (red fluorescence), lateral (green fluorescence) and medial + lateral (red and green fluorescence) axons. **f** When comparing tadpoles treated with DSCAM MO vs Control MO (targeted to ventral RGCs), no difference in the area occupied by arbors from ventral RGCs that project medially or regions occupied by dorsal RGCs axons that project laterally. Statistical analysis was by unpaired, two-tailed t test with equal sample sizes (*n* = 8) for Control MO and DSCAM MO. Error bars indicate S.E.M. *** *p* ≤ 0.001, **** *p* ≤ 0.0001, *ns* = non-significant. Scale bars: 40 μm

Analysis of axon terminals along the lateral-medial axis (as depicted schematically in Fig. 6a), showed that ventral RGC axons (labeled with lissamine-tagged MO) innervate the tectum medially, while dorsal RGC axons (labeled with Alexa 488 dextran) travel more laterally, as shown for other species [9, 35]. Indeed, ventral and dorsal RGC axons from tadpoles injected at stage 46 and imaged 48 h later (see Fig. 6b) showed correct topographic mapping but with a consistent degree of arbor overlap as shown in Fig. 6d. When retinal neurons were labeled at a later stage, at stage 47 and imaged 48 h after, medial arbors were visibly separated from lateral arbors (Fig. 6c). This separation between lateral and medial arbors in the *Xenopus* tadpole is consistent with observations in zebrafish larvae at 5 days postfertilization, when the optic tectum is first fully innervated [9]. Thus these in vivo imaging studies confirm that in *Xenopus,* dorsal RGC axons projecting through the lateral branch initially overlap with ventral RGC axons traveling through the medial branch; then, as the tectum expands and arbors become more complex, laterally and medially projecting arbors remodel and clearly separate along the *Xenopus* neuropil.

To identify specific cellular actions of DSCAM in directing retinotopy in the tectum, we targeted the population of RGCs that preferentially express DSCAM to manipulate its expression at the time when a majority of axons have already arborized in the tectum, but when medially and laterally projecting axons still overlap. For this, we electroporated a morpholino (MO) targeting *Xenopus laevis Dscam* mRNA to block translation and downregulate endogenous DSCAM levels in axons of ventral RGCs in tadpoles at stage 46, while also labeling dorsal axons with Alexa 488 dextran. This strategy allowed us to manipulate and visualize the innervation patterns and topographic organization of axon arbors in the neuropil rather than interfere with axon pathfinding or initial axon branching [23]. As shown for control tadpoles, axons derived from ventral and dorsal RGCs were correctly sorted along the medial–lateral axis (Fig. 6d), with ventral RGC axons predominantly arborizing in the medial portion of the neuropil and dorsal RGCs axons arborizing laterally. However, 48 h after DSCAM MO injection, ventral RGC axon arbors seemed to be positioned more medially compared to controls (Fig. 6d). To quantify this effect, we measured the area occupied by the axon arbors within the tectal neuropil; total arbor area (in pixels) from ventral RGCs injected with DSCAM MO was compared to that from ventral RGCs in sibling tadpoles injected with control MO. The average arbor spread of axons positioned medially in tadpoles with DSCAM MO knockdown was not significantly different from controls (Fig. 6f). Dorsal RGC axons labeled with Alexa 488 dextran projecting laterally within the tectum in either control MO or DSCAM MO treated tadpoles also occupied a similar area independent of ventral RGC treatment (Fig. 6f). As shown above, in stage 46 tadpoles there is a degree of overlap between medially-projecting ventral RGC axons (red fluorescence) and laterally-projecting dorsal RGC axons (green fluorescence) within the tectal neuropil (Fig. 6d). When calculating the area of overlap occupied by dorsal (Alexa 488-dextran) and ventral RGC (lissamine-tagged MO) axons in the same tadpoles (normalized to percent of total area), DSCAM MO knockdown showed a significant reduction in arbor overlap compared to the overlap of arbors in control MO treated tadpoles (Fig. 6e). This difference was significant when analyzing overlap in relation to the area extent of the medially projecting ventral RGC axons (red fluorescence; Control MO 47.50 ± 2.7%, *n* = 8; DSCAM MO 24.55 ± 2.5%, *n* = 8, *p* ≤ 0.0001), laterally projecting dorsal RGC axons (green fluorescence; Control MO 44.49 ± 4.3%; DSCAM MO 23.20 ± 2.3%, *p* = 0.0004) and the combined projection of medial and laterally projecting axons (red and green fluorescence; Control MO 24.04 ± 1.1%; DSCAM MO 11.87 ± 1.1%, *p* ≤ 0.0001). Together, these findings suggest that changes in DSCAM expression in ventral RGC axons affect their projection patterns at the target, where an increase in segregation of medial and lateral axons is observed in response to lowered endogenous DSCAM levels.

### Dendritic localization of DSCAM in tectal neurons

**Fig. 7:**
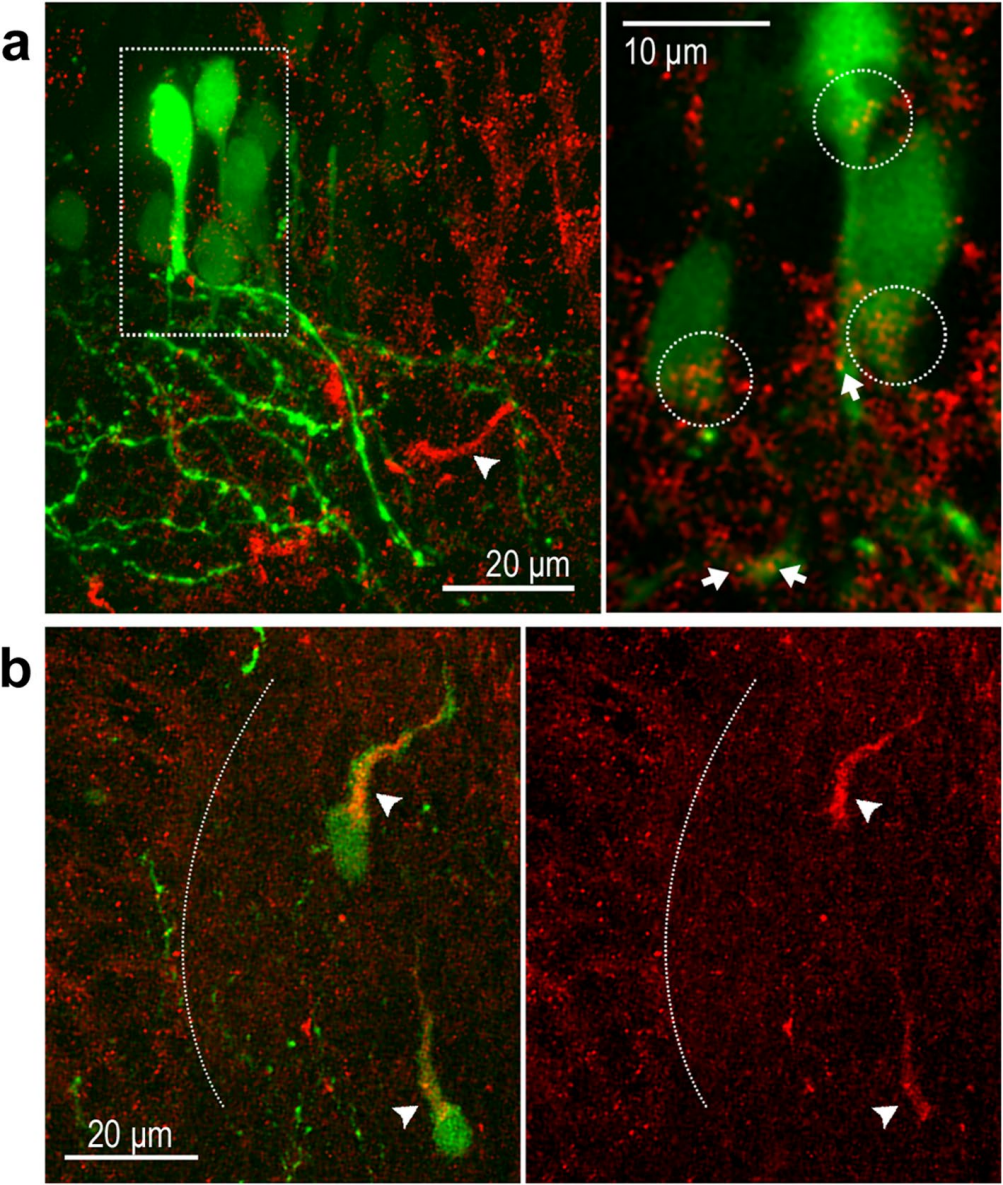
Localized DSCAM immunoreactivity to primary process and cell bodies within the tectum and tectal neuropil. Electroporation with a CMV-driven GFP expression plasmid was used to randomly label neurons in young embryos. Coronal sections from stage 46 tadpoles with GFP-positive cells were immunostained for DSCAM. **a** *Left panel:* A maximum confocal projection at the level of the midbrain shows GFP positive neurons immunostained for DSCAM. *Right panel:* A large magnification of a single confocal plane (*white box*; left panel), shows punctate DSCAM immunoreactivity localized on cell bodies of tectal neurons (*white dotted circles*) as well as on primary dendrites and dendritic branches (*white arrows*). Note that strong DSCAM immunolabeled fibers are present near dendrites of GFP labeled neurons, as shown in the low magnification image (*arrowhead*). **b** Analysis of tissues with low-yield, random GFP transfection revealed that strong DSCAM immunoreactivity localizes to primary processes of GFP-expressing simple cells within the tectal neuropil (*arrowhead*). The white dotted line indicates the boundary between the cell body layer and the neuropil. Scale bars: 20 μm for a and b; 10 μm for the magnified view of panel a

Our previous work showed that downregulation of DSCAM expression in single RGCs interferes with axon growth and branching at the target, indicating that endogenous DSCAM acts as permissive cue that facilitates RGC axon growth. In contrast, single-cell downregulation or overexpression of DSCAM in tectal neurons showed that DSCAM acts as a restrictive cue to regulate the size and complexity of their dendritic arbors [23]. Thus, in addition to RGCs, DSCAM can differentially influence postsynaptic neurons in the *Xenopus* visual system. Indeed, punctate DSCAM immunoreactivity can be detected not only within the *Xenopus* retina but also surrounding cell bodies in the tectum as well as in the tectal neuropil in unpermeabilized tissues (Fig. 7a, see also [23]). Analysis of tissues further revealed a unique pattern DSCAM immunoreactivity, with the DSCAM antibody strongly labeling thin processes within the tectal neuropil (Fig. 7a, arrowheads). To further characterize DSCAM expression, we electroporated embryos with a GFP plasmid at low concentration to randomly label cells in the brain. At stage 45, tadpoles were screened for the presence of isolated or small clusters of GFP-expressing neurons and were fixed and immunostained for DSCAM. DSCAM immunoreactivity localized to cell bodies, primary dendrites and dendritic branches of GFP-expressing tectal neurons (white arrows Fig. 7a). The random transfection and expression of GFP within the brain also revealed strong DSCAM immunoreactivity on primary processes of GFP-expressing cells that were positioned within the neuropil (Fig. 7b). The identity of these cells in *Xenopus* is unknown, but they share similar morphology and features to tegmental projection neurons characterized in id2b transgenic zebrafish larvae that are found exclusively in the neuropil and have a prominent primary process that protrudes apically [36]. Thus, these experiments confirm specific localization of DSCAM not only on RGC axons but also on tectal neurons and raise the possibility of additional potential roles for DSCAM in neurons within the neuropil.

## Discussion

Our previous studies explored cell-autonomous roles for DSCAM during the development of pre- and postsynaptic structural and functional connectivity in the developing *Xenopus* retinotectal circuit. We found that DSCAM primarily acts as a neuronal brake to limit and guide postsynaptic dendrite growth of tectal neurons while it also facilitates arborization of presynaptic RGC axons cell autonomously [23]. In that study, we targeted ventral RGCs for our analysis since their axons are easier to visualize in vivo with confocal imaging as they project to the most dorsal part of the tectal neuropil [37]. For this study, we characterized the expression of DSCAM along the ventrodorsal axis of the optic nerve, and we followed the navigation of ventral and dorsal retinal axons corresponding to this expression. We found a specific pattern of DSCAM expression along the *Xenopus* optic nerve that correlated with how optic nerve fibers are topographically organized. Fasciculated bundles of ventral fibers derived from ventral RGCs normally navigate the optic nerve along its ventral side, which coincided with strong DSCAM expression. After retinal fibers crossed the optic chiasm to project dorsally, we observed that DSCAM immunoreactivity became less distinguishable in the RGC axon bundles as they traveled along the optic tract, where punctate DSCAM immunoreactivity throughout the brain tissue is present. This dispersion of axon bundles coincides with ventral and dorsal retinal axons rearranging topographically as fibers pass the chiasm and project contralaterally into the optic tract and optic tectum. DSCAM has been well characterized as a homophilic binding molecule mediating intracellular adhesion and the fasciculation of axon bundles [15, 38]. The site and timing of expression suggests that DSCAM is involved, to some degree, in maintaining the ventrodorsal topography of optic nerve fibers and the spatial arrangement that mirrors how axons exit the optic nerve head. It is possible that through its adhesive properties and homophilic interactions, DSCAM serves to anchor ventral fibers together, preventing any rearrangement or interchange with dorsal axons as fibers navigate the optic pathway from the optic nerve head to the chiasm. Differential fasciculation of fibers along the optic nerve may be an underlying mechanism to traffic axons in an orderly manner to the chiasm [15]. Organized arrival of axons at the site of the chiasm would allow axons to respond to the next set of guidance cues, including ephrins, other chemoattractant cues, and neurotrophic factors [28, 39–42], which all prepare for the subsequent stage of morphological trajectory into the tectum. We showed preferential DSCAM expression on ventral RGC axons and along the posterior region of the *Xenopus* optic chiasm as well (Fig. 2a), a finding that is in agreement with observations of DSCAM expression in the posterior region of the mouse optic chiasm [15].

In addition to mechanisms organizing the topography and spatial arrangement of axon fibers, it is important to note that there are also time-based mechanisms involved that indirectly contribute to the topographic wiring of circuits. During *Xenopus* eye development, new retinal ganglion cells are generated at the ciliary margin located at the periphery of the eye [37, 43]. Older cells are pushed towards the central portion of the retina and a gradient of maturing cells is created along the retina radius. Because of the temporal pattern of early eye development, the deployment of emerging RGC axons along the optic pathway is set to a defined temporal sequence. Dorsal retinal fibers exit the eye first, navigate the optic pathway, and reach the tectum six hours ahead of ventral retinal axons. The newer set of axon fibers exiting the eye travel along the most ventral portion of the optic nerve as innervation takes place [37, 44]. It is possible that fasciculation of retinal fibers by DSCAM indirectly modulates the pacing of “younger” ventral axons along the optic nerve – perpetuating a difference in timing at which ventral and dorsal axons reach their target sites. In our previous work using real-time imaging of RGC axons as they innervate the optic tectum, we showed that DSCAM is important in promoting the branching rate of retinal axons in vivo [23], which supports the idea that DSCAM is involved in distinct temporal aspects of RGC axon development and differentiation.

Differential timing of retinotectal projections was initially thought to be the mechanism that generates topographic mapping in the optic tectum, with the argument that pioneering dorsal fibers innervate ventral areas in the tectum simply for arriving first at the available sites. This hypothesis stated that ventral fibers of the retina would later follow and would be forced to occupy the next available sites at the dorsal area of the tectum, due to the constraints of existing dorsal axons [37]. Studies, however, have shown that disrupting the timing of retinal axon deployment, by heterochronic transplantation of early age RGCs into older embryos, does not seem to affect the topographic mapping formed during development, indicating that other mechanisms are at work [37]. It is becoming increasingly evident, based on a number of studies, that sub-populations of RGCs employ different molecular and cellular strategies to achieve axon-target specificity [45, 46]. For example, sub-populations of RGCs heavily rely on repellant and attractive cues for precise axon targeting. In amphibians, populations of RGCs differentially express ephrin-Bs in a high dorsal to low ventral gradient in the retina [47, 48]. This gradient pattern in the retina complements EphB1 receptors expression along the *Xenopus* tectum which is distributed in a high ventral to low dorsal gradient. Signaling between EphB1 receptors and ephrin-B ligands have been suggested to be the underlying mechanism that attracts dorsal retinal axons into the ventral portion of the tectum [47]. The work we present in this study adds DSCAM to a growing of list of molecular strategies that retinal axons may use to self-organize topographically along the optic nerve and within the target.

We observed that DSCAM immunoreactivity is more diffuse after axons cross the optic chiasm and enter the optic tract where other cell types express DSCAM, but then increases gradually at the level of the tectal neuropil where retinal axons arborize and form connections with post-synaptic tectal partners. At the spatial gap within the optic tract where DSCAM expression is lower, axons fibers are rearranged, most likely by pre- and post-synaptic molecular interactions mediated by Ephs/ephrin signaling, to reorient the topography of axons in an inverted manner, while also distributing their projection along the mediolateral axis to enter the neuropil. The relative increase in DSCAM immunoreactivity at the site where RGC axons terminate (as visualized by the 3A10 staining) may signify not only the localized expression of DSCAM in retinal axon terminals but also in tectal neuron dendrites that project to and actively branch within the tectal neuropil. Here we tested, separately from its effect on axon branching, whether DSCAM plays a role in the arrangement of arbors across the mediolateral plane of the tectal neuropil. We examined effects of DSCAM on ventral RGCs, the same population of RGCs examined in our previous studies [23], but targeted DSCAM MO knockdown through retinal electroporation in tadpoles at later stages. We found that downregulating DSCAM expression in ventral RGCs with axons already terminating medially within the tectum caused a shift and extension of their terminal arbors away from those of dorsal RGCs, suggesting that DSCAM guides remodeling and topographic organization of arbors derived from ventral RGCs. During zebrafish development (which closely resembles *Xenopus* development), dorsal retinal fibers normally reach the optic tectum via the lateral branch, while ventral axons project via the medial branch [9]. Disrupting mechanisms dependent on RNA-binding proteins, such as Hermes that modulates guidance cue receptor expression, causes an aberrant shift in topographic ordering and results in lateral dorsal axons projecting ectopically into the medial branch arbor [9]. Thus, our studies indicate that DSCAM, together with other guidance and signaling molecules, participates in the medio-lateral topographic mapping at the target.

During RGC axon arborization, coordinated addition and retraction of axonal branches and of dendrites of tectal neurons allows for a gradual recognition between pre- and postsynaptic partners which allows for new synaptic connections to be formed [49, 50]. Additionally, bi-directional communication at the molecular level is also thought to be at work facilitating synaptogenesis. For example, neurotrophins, including brain-derived neurotrophic factor (BDNF), can act as a retrograde signal to influence presynaptic neurons, while also acting as an anterograde factor on postsynaptic cells [49, 51]. This type of bi-directional signaling can generally induce the development and maturation of synapses, or even modify the structure of existing synapses. Unpublished work from our laboratory shows that DSCAM localizes to only a sub-set of retinotectal synapses, suggesting that endogenous DSCAM, localized post-synaptically may be implicated in the stability and/or maintenance of synapses (R.A. Santos and S. Cohen-Cory, unpublished). Studies have shown that topographic arrangement of axons is also precisely organized at the synapse level. Studies both in mouse and in *C. elegans* indicate that graded inhibitory cues for synapse formation and maintenance are also used to restrict synapse distribution and create synapse topographic maps [52]. Homophilic binding between DSCAM proteins in rodents mediates neurite adhesion, which helps facilitate precise synaptic targeting within a specific sub-lamina in the retina [29]. DSCAM can also functionally interact with other cell-adhesion molecules, specifically cadherins and protocadherins, to “mask” their adhesive properties and consequently prevent neurite collision and fasciculation [53]. In Aplysia, DSCAM acts trans-synaptically and in collaboration with AMPA-like receptors promotes synapse formation [54]. In the developing *Xenopus* tadpole, visually driven Ca^2+^ signals are topographically organized at the subcellular dendritic scale in tectal neurons [35]. Characterizing the spatial distribution of molecules, such as DSCAM, on both pre- and post-synaptic arbors to match their anatomical location along synapses remains open to investigation.

## Conclusion

In *Xenopus*, endogenous DSCAM acts at multiple levels along the visual circuit, independently modulating dendrite and axon arborization, where cell-autonomous roles vary depending on the cell type [23]. Our current work demonstrates that DSCAM is also involved in the organization of axon terminals at the target and that these cell-autonomous effects correlate with differential DSCAM expression along the retinotectal pathway. Potential roles for DSCAM in directing the organization of *Xenopus* retinal axons as they travel along the optic nerve and chiasm, in sorting and remodeling of axon arbors along a topographic axis within the neuropil, and in maintaining pre- and postsynaptic retinotectal arbors are consistent with those in mammals [15], and support multiple roles for DSCAM during vertebrate neural circuit development.

## Data Availability

The datasets used and/or analyzed during the current study are available from the corresponding author on reasonable request.

## Abbreviations

CNS: Central nervous system
DSCAM: Down syndrome cell-adhesion molecule
GCL: Ganglion cell layer
INL: Inner nuclear layer
IPL: Inner plexiform layer
MO: Morpholino antisense oligonucleotide
RGC: Retinal ganglion cell
